# Therapy response in seronegative versus seropositive autoimmune encephalitis

**DOI:** 10.3389/fimmu.2023.1196110

**Published:** 2023-05-31

**Authors:** Benjamin Berger, Sophie Hauck, Kimon Runge, Ludger Tebartz van Elst, Sebastian Rauer, Dominique Endres

**Affiliations:** ^1^Clinic of Neurology and Neurophysiology, Medical Center - University of Freiburg, Faculty of Medicine, University of Freiburg, Freiburg, Germany; ^2^Department of Neurology, Helios Clinic Pforzheim, Pforzheim, Germany; ^3^Department of Psychiatry and Psychotherapy, Medical Center - University of Freiburg, Faculty of Medicine, University of Freiburg, Freiburg, Germany

**Keywords:** autoimmune encephalitis, antibody, seronegative, immunotherapy, diagnostic criteria

## Abstract

**Background:**

Autoimmune encephalitis (AE) might be seropositive or seronegative, depending on whether antibodies targeting well-characterized neuronal antigens can be detected or not. Since data on treatment efficacy in seronegative cases, are scarce, the main rationale of this study was to evaluate immunotherapy response in seronegative AE in comparison to seropositive cases.

**Methods:**

An electronic database search retrospectively identified 150 AE patients, treated in our tertiary care university hospital between 2010 and 2020 with an AE. Therapy response was measured using both general impression and the modified Rankin Scale (mRS).

**Results:**

Seventy-four AE patients (49.3%) were seronegative and 76 (50.7%) seropositive. These cases were followed up for a mean of 15.3 (standard deviation, SD, 24.9) and 24.3 months (SD 28.1), respectively. Both groups were largely similar on the basis of numerous clinical and paraclinical findings including cerebrospinal fluid, electroencephalography, magnetic resonance imaging, and 18-F-fluor-desoxy-glucose-positron-emmission-tomography pathologies. The majority of patients (80.4%) received at least one immunotherapy, which were glucocorticoids in most cases (76.4%). Therapy response on general impression was high with 49 (92.5%) of treated seronegative, and 57 (86.4%) of treated seropositive AE cases showing improvement following immunotherapies and not significantly different between both groups. Notably, the proportion of patients with a favorable neurological deficit (mRS 0-2) was twice as high during long-term follow-up as compared to baseline in both groups.

**Conclusion:**

Since both, patients with seronegative and seropositive AE, substantially benefitted from immunotherapies, these should be considered in AE patients irrespective of their antibody results.

## Highlights

*What is already known on this topic*

Data on treatment response in patients with seronegative autoimmune encephalitis (AE), particularly in comparison to seropositive cases, are scarce.

*What this study adds*

Our study demonstrates that the vast majority of both patients with seronegative and seropositive AE benefitted from immunotherapies.

*How does this study affect research, practice or policy*

Immunotherapies should be considered in patients with AE, regardless whether they are seronegative or seropositive.

## Introduction

Autoimmune encephalitis (AE) is a rare immune-mediated disorder of the brain (1) that commonly presents with neurologic or psychiatric symptoms (e.g., cognitive deficits, abnormal movements, seizures, changes in behavior, psychosis, or decreased level of consciousness) (2). In many cases, immunoglobulin (Ig) G antibodies that target either well-characterized neuronal cell-surface or intracellular antigens can be detected. Antibodies targeting cell-surface antigens are pathophysiologically relevant, and in some cases malignancies or preceding viral infections trigger the immune process (2). In comparison, antibodies directed against intracellular antigens are an epiphenomenon of the immune process, which is mediated by CD8+ cytotoxic T cells, and these conditions are in the vast majority of cases paraneoplastic, i.e., associated with cancer (2–4). Patients suffering from AE and with well-characterized antibodies targeting either cell-surface or intracellular antigens are classified as seropositive (5). In contrast, seronegative AE accounts for cases, in which no antibodies are detectable. These might comprise patients with neuronal antibodies against yet to be identified antigens as well as patients with cell-mediated immune processes (5, 6).

Treatment of AE includes immunotherapy as well as elimination of a potential trigger, e.g., removal of an underlying malignancy (2). Despite the lack of randomized, controlled trials, there is an expert recommendation that immunotherapy includes glucocorticoids, intravenous immunoglobulins (IVIG), or plasma exchange as first-line, followed by longer-term immunosuppression (e.g., with rituximab) in therapy refractory cases and/or to prevent relapses as second-line (2, 7–9). Many patients are initially severely affected, but remarkably improve with immunotherapies (7). In this regard, time until start of treatment substantially affects prognosis, with early initiation and consistent escalation resulting in the best prognosis (7). Obviously, diagnostic uncertainty in seronegative AE cases might result in treatment delay and less aggressive therapy, which in turn results in poorer therapy response (10). Hence, early recognition of these patients, accurate diagnosis, and treatment initiation are crucial (7). Of course, differential diagnoses, particularly infectious origins, have to be excluded beforehand (5).

In 2016, Graus and colleagues published international consensus criteria for the diagnosis of AE based on clinical aspects (subacute onset of short-term memory loss, altered mental status, seizures, or psychiatric symptoms), standard diagnostic tests (e.g., magnetic resonance imaging (MRI), electroencephalography (EEG), or cerebrospinal fluid (CSF) studies) as well as antibody testing. As a key aspect of these criteria, patients might be diagnosed with AE even in the absence of antibodies, e.g., before test results are available or if tests are negative for well-characterized antibodies. Based on the criteria, patients might be diagnosed with “possible”, “probable”, or “definite” AE (5). Although differential diagnoses have to be excluded, the terms “probable” and “possible” in seronegative patients implied that some patients might be misdiagnosed as having AE. In this regard, diagnostic criteria have not been validated yet. In an ideal scenario, the majority of patients diagnosed with AE should benefit from immunotherapies if there is an underlying autoimmune cause.

Therefore, the main rationale of this study was to evaluate response to immunotherapies in patients diagnosed with seronegative AE in comparison with seropositive cases. Secondary, both groups were compared regarding clinical and paraclinical findings.

## Methods

The electronic database of our hospital was retrospectively screened for neurologic or psychiatric patients, who had been treated between 2010 and 2020, and who had been diagnosed with the ICD-10 codes G04.8 (neurology) and F06.- (psychiatry), respectively. Cases were included if they were fulfilling current diagnostic criteria for definite, probable or possible AE (5) according to data obtained from their electronic records. Patient identification and verification of diagnosis according to these criteria was done by one trained investigator (SH). All cases not fulfilling consensus criteria, were discussed together with a senior neurologist (BB) based on recently published recommendations for diagnosis and management of AE (9). Patients with alternative causes of encephalitis/encephalopathy (e.g., infectious, metabolic, endocrine, psychiatric, or rheumatic disease) were excluded.

According to their antibody status patients were classified as either seropositive, if they had well-characterized antibodies in serum and/or cerebrospinal fluid (CSF), or seronegative, if no antibodies had been detected. Well-characterized antibodies included those against cell-surface (N-methyl-D-aspartate receptor (NMDAR), α-amino-3-hydroxy-5-methyl-4-isoxazolepropionic receptor (AMPAR) 1 or 2, dipeptidyl-peptidase-like protein-6 (DPPX), leucine-rich glioma inactivated 1 (LGI1), contactin-associated protein-2 (CASPR2), γ-aminobutyric acid receptor (GABA) A or B, glycine receptor, IgLON-5, and metabotropic glutamate receptor (mGluR) 1 or 5) or intracellular antigens (Hu, Yo, Ri, CV2 (CRMP5), amphiphysin, Ma1, Ma2, SOX1, Tr (DNER), Zic4, GAD65). In these cases, antibody detection had been performed by commercially available assays (cell-surface antibodies: Euroimmun^®^, Lübeck, Germany; intracellular onconeural antigens: ravo Diagnostika^®^, Freiburg, Germany) according to the manufacturers’ instructions. In addition, two patients with antibodies targeting neurochondrin and Nb/AP3B2 (adaptor protein-3B2), respectively, were included, since these are associated with well-characterized, autoimmune-mediated clinical syndromes, i.e., non-paraneoplastic cerebellar degeneration (11–14). These antibodies were detected by immunofluorescence in an external referral laboratory (Laboratory Stoecker, Gross Groenau, Germany).

All patients’ demographic, clinical, and paraclinical data were obtained from electronic records. Therapy response was measured using both “general impression”, which had been documented as a qualitative, non-standardized measure by the treating physician at the time of the clinical visit, and the modified Rankin Scale (mRS), which was estimated retrospectively.

For comparison of seronegative and seropositive AE patients, categorical variables are presented using numbers and percentages; continuous variables are presented using means, range, and standard deviation (SD). Statistical analyses were performed using Fishers exact test or Chi square test for categorical variables. T-test for independent samples or Mann-Whitney U test were used for continuous variables depending on whether the data were normally distributed or not (analyzed using Kolmogorov-Smirnov test). A p value < 0.05 was regarded as statistically significant, and a p value < 0.10 was described as a trend. For all statistical analyses SPSS (version 28) software (IBM^®^, Armonk, USA) was used.

The study was approved by the local ethics committee of the University of Freiburg (No. 20-1018).

## Results

The electronic database search identified 150 patients with AE. One hundred and eighteen (78.7%) were fulfilling consensus criteria. Of these, 22 (14.7%) had definitive seropositive AE, 18 (12.0%) definitive NMDAR encephalitis, 36 (24.0%) definitive limbic encephalitis (29 seropositive, seven seronegative), 39 (26.0%) possible and three (2.0%) probable AE. Thirty-two (21.3%) were not strictly fulfilling consensus criteria, but were still included, since they had the clinical picture of AE and extensive exclusion of alternative diagnosis. Though 10 (6.7%) presented outside the subacute stage of the disease of three months, and the others had paraclinical findings indicating an inflammatory etiology not covered by consensus criteria: 16 (10.7%) had antibodies against unknown neuronal antigens, five (3.3%) had 18-F-fluor-desoxy-glucose-positron-emmission-tomography (FDG-PET) results clearly indicative of an inflammatory etiology, and one (0.7%) had CSF-restricted oligoclonal bands (OCB), but not pleocytosis on CSF analysis.

### Patient characteristics

**Table 1** summarizes various demographic, serological, and clinical features. Seventy-four patients (49.3%) were seronegative, whereas 76 (50.7%) had at least one well-characterized antineuronal antibody (therefore seropositive). Age and sex were well-balanced between the groups. As expected, the majority of patients with seropositive AE had antibodies targeting cell-surface antigens, mostly NMDAR antibodies (in 21 cases) and LGI1 antibodies (in 19 patients). Even though there was no significant difference in the overall number of malignancies between the groups, bronchial carcinomas were less frequent in the seronegative AE group (1 (1.4%) *vs* 8 (10.5%) cases, p = 0.034). As clinical manifestations, epileptic seizures (26 (36.1%) *vs* 41 (54.0%), p = 0.029), and focal neurological deficits (18 (24.7%) *vs* 31 (40.8%), p = 0.036) were less frequent in the seronegative AE group, whereas other symptoms were equally distributed. Patients with seronegative AE were statistically significantly more severely affected at the time of admission as measured by the modified Rankin Scale (mean mRS 3.3 ± 1.1 *vs* 2.9 ± 1.0, p = 0.038).

**Table 1:** Demographics, antibody status, malignancy, clinical features, and baseline clinical parameters of patients with seronegative and seropositive AE.

	All n=150	Seronegative AE n=74	Seropositive AE n=76	p value (seronegative *vs*. seropositive)
Demographic parameters				
Age in years at primary manifestation, *mean (range, SD)*	49.5 (15-87; 20.2)	50.0 (15-87; 22.2)	48.9 (19-81; 18.1)	T=0.354, p_t-test_=0.724
Age in years at diagnosis, *mean (range, SD)*	50.8 (17-87; 19.5)	51.4 (17-87; 21.3)	50.2 (19-81; 17.8)	T=0.372, p_t-test_=0.710
Females*, n (%)*	78 (52.0%)	35 (47.3%)	43 (56.6%)	Chi²=1.294, p=0.255
Males*, n (%)*	72 (48.0%)	39 (52.7%)	33 (43.4%)	
Antibody status*, *n (%)*				
One anti-neuronal antibody	66 (44.0%)	0	66 (86.8%)	–
Multiple antibodies (≥ 2)	10 (6.7%)	0	10 (13.2%)	–
Neuronal IgG cell surface antibodies*	52 (34.7%)	0	52 (68.4%)	–
NMDAR	21 (14.0%)	0	21 (27.6%)	–
LGI1	19 (12.7%)	0	19 (25%)	–
CASPR2	5 (3.3%)	0	5 (6.6%)	–
GABA_B_	4 (2.7%)	0	4 (5.3%)	–
GlyR	2 (1.3%)	0	2 (2.6%)	–
AMPAR	1 (0.7%)	0	1 (1.3%)	–
DPPX	0 (0.0%)	0	0 (0.0%)	–
IgLON5	0 (0.0%)	0	0 (0.0%)	–
mGluR1	0 (0.0%)	0	0 (0.0%)	–
mGluR5	0 (0.0%)	0	0 (0.0%)	–
Intracellular/synaptic neuronal IgG antibodies*	35 (23.3%)	0	35 (46.1%)	–
Hu/ANNA-1	7 (4.7%)	0	7 (9.2%)	–
Yo/PCA-1	2 (1.3%)	0	2 (2.6%)	–
CV2/CRMP5	2 (1.3%)	0	2 (2.6%)	–
Ri/ANNA-2	1 (0.7%)	0	1 (1.3%)	–
Ma1	1 (0.7%)	0	1 (1.3%)	–
Ma2 (Ta)	0 (0.0%)	0	0 (0.0%)	–
Amphiphysin	1 (0.7%)	0	1 (1.3%)	–
GAD65	10 (6.7%)	0	10 (13.2%)	–
SOX1/AGNA	7 (4.7%)	0	7 (9.2%)	–
Zic4	2 (1.3%)	0	2 (2.6%)	–
Neurochondrin	1 (0.7%)	0	1 (1.3%)	–
Nb/AP3B2	1 (0.7%)	0	1 (1.3%)	–
Baseline clinical parameters n=149				
Time in weeks from primary manifestation to hospital admission, *mean (range, SD)* *n=140*	15.5 (0-468; 46.5)	14.0 (0-130; 27.3)	16.9 (0-468; 59.0)	T=-0.366, p_t-test_=0.715
Modified Rankin Scale (mRS), *mean (range, SD)*	3.1 (1-5; 1.0)	3.3 (1-5; 1.1)	2.9 (1-5; 1.0)	**T=2.096,** **p_t-test_=0.038**
mRS 0-2, *n (%)*	41 (27.5%)	16 (21.9%)	25 (32.9%)	Chi²=2.249, p=0.134
mRS 3-6, *n (%)*	108 (72.5%)	57 (78.1%)	51 (67.1%)	
Clinical features*, n (%)* n=149				
Epileptic seizures n=148	67 (45.3%)	26 (36.1%)	41 (54.0%)	**Chi²=4.747, p=0.029**
Reduced vigilance n=146	34 (23.3%)	16 (22.9%)	18 (23.7%)	Chi²=0.014, p=0.906
Psychiatric symptoms	82 (55.0%)	43 (58.9%)	39 (51.3%)	Chi²=0.866, p=0.352
Memory problems	113 (75.3%)	56 (75.7%)	57 (75.0%)	Chi²=0.009, p=0.924
Focal neurological deficits	49 (32.9%)	18 (24.7%)	31 (40.8%)	**Chi²=4.390, p=0.036**
Autonomic dysregulation	6 (4.0%)	2 (2.7%)	4 (5.3%)	p_Fisher_=0.681
Malignancy, *n (%)* n=149				
Bronchial carcinoma	9 (6.0%)	1 (1.4%)	8 (10.5%)	**p_Fisher_=0.034**
Bronchial carcinoid	1 (0.7%)	0 (0.0%)	1 (1.3%)	
Breast cancer	5 (3.4%)	1 (1.4%)	4 (5.3%)	p_Fisher_=0.367
Cervical cancer	1 (0.7%)	0 (0.0%)	1 (1.3%)	
Prostate cancer	2 (1.3%)	2 (2.7%)	0 (0.0%)	
Renal cell carcinoma	1 (0.7%)	1 (1.4%)	0 (0.0%)	
Basal cell carcinoma	1 (0.7%)	2 (2.7%)	0 (0.0%)	
Multiple myeloma	2 (1.3%)	2 (2.7%)	0 (0.0%)	
Hypopharynx carcinoma	1 (0.7%)	1 (1.4%)	0 (0.0%)	
Teratoma	1 (0.7%)	0 (0.0%)	1 (1.3%)	
**Overall malignant tumors,** ***n (%)***	26 (17.4%)	10 (13.7%)	16 (21.1%)	Chi²=1.398, p=0.237

*Patients with uncharacterized novel antibodies (e.g., against vessels) were classified as seronegative AE.

AE, autoimmune encephalitis; AMPAR, α-amino-3-hydroxy-5-methyl-4-isoxazolepropionic receptor 1 or 2; CASPR2, contactin-associated protein-2; CV2/CRMP5,collapsin response mediator protein 5; DPPX, dipeptidyl-peptidase-like protein-6; GABAB, γ-aminobutyric acid receptor B; GAD65, glutamic acid decarboxylase 65; GlyR, glycine receptor; Hu/ANNA-1, antineuronal nuclear autoantibody type 1; IgG, immunglobulin G; IgLON5, immunglobulin-like cell adhesion molecule 5; LGI1, leucine-rich glioma inactivated 1; mGluR1, metabotropic glutamate receptor 1; mGluR5, metabotropic glutamate receptor 5; mRS, modified Ranking Scale; Nb/AP3B2, β-neuronal adaptin-like protein; NMDAR, N-methyl-D-aspartate receptor; Ri/ANNA-2, antineuronal nuclear autoantibody type 2; SD, standard deviation; SOX1/AGNA, SRY-Box Transcription Factor 1/anti-glia nuclear antibody; Yo/PCA1, Purkinje cell cytoplasmatic antibody type 1; Zic4, zinc-finger-protein-4.

Significant results are typed in bold.

### Paraclinical findings

Regarding CSF parameters, there was a trend towards CSF-restricted OCBs being less frequent in the seronegative group (12 (17.1%) *vs* 21 (30.9%) in the seropositive AE cases, p = 0.059), whereas an increased albumin quotient as a marker of blood-CSF barrier dysfunction was significantly more frequent (seronegative 31 (43.1%) *vs* seropositive 19 (26.8%), p = 0.041). Various abnormalities on EEG were balanced between the groups. The proportion of patients with abnormal brain MRI (seronegative 57 (80.3%) *vs* seropositive 52 (74.3%) cases, p = 0.395), and FDG-PET (seronegative 50 (86.2%) *vs* seropositive 63 (91.3%) patients, p = 0.361) was not statistically different between the groups. The high rate of FDG-PET investigations (127/150 patients (84,7%)) is attributable to the generally low-threshold use of this modality in our hospital, irrespective of MRI results. In addition, most patients received both FDG-PET of the brain and the whole body; the latter for screening of malignancies. More details on CSF parameters, EEG findings, and imaging results are presented in **Table 2**.

**Table 2:** Cerebrospinal fluid parameters, electroencephalography pathologies, and brain imaging findings of patients with seronegative and seropositive AE.

	All n=150	Seronegative AE n=74	Seropositive AE n=76	p value (seronegative *vs*. seropositive)	
CSF parameters, n (%) n=145					
Pathological CSF alterations overall, *n (%)*	89 (61.4%)	47 (64.4%)	42 (58.3%)	Chi²=0.560, p=0.454
White blood cell count (/µl), *mean (range, SD)* *n=143*	12.1 (1-197; 29.7)	11.5 (1-176; 30.4)	12.6 (1-197; 29.2)	T=-0.219, p_t-test_=0.827
Increased cell count (ref. < 5/µl) n=143	48 (33.6%)	20 (27.8%)	28 (39.4%)	Chi²=2.179, p=0.140
Protein concentration (mg/l), *mean (range, SD)* n=143	595.0 (109-3090; 443.4)	607.0 (157-3090; 457.5)	582.8 (109-2760; 431.5)	T=0.325, p_t-test_=0.745
Increased protein concentration (ref. < 450 mg/l) n=143	80 (55.9%)	40 (55.6%)	40 (56.3%)	Chi²=0.009, p=0.925
Albumin quotient, *mean (range, SD)* n=143	9.1 (1.8-80.3; 9.3)	9.7 (1.8-80.3; 11.0)	8.5 (1.9-38.7; 7.2)	T=0.716, p_t-test_=0.475
Increased albumin quotients (ref.: <40y.: < 6.5 x 10^-3^; 40-60y.: < 8 x 10^-3^; >60y.: < 9.3 x 10^-3^) n=143	50 (35.0%)	31 (43.1%)	19 (26.8%)	**Chi²=4.174, p=0.041**
CSF-restricted OCBs, *n (%)* n = 138	33 (23.9%)	12 (17.1%)	21 (30.9%)	**Chi²=3.579, p=0.059**
Increased IgG-Index (ref. < 0.7) n=143	22 (15.4%)	8 (11.1%)	14 (19.7%)	Chi²=2.034, p=0.154
EEG findings, *n (%)* n=129					
Pathological EEG overall	74 (57.4%)	41 (60.3%)	33 (54.1%)	Chi²=0.505, p=0.477
Generalized slowing	73 (56.6%)	41 (60.3%)	32 (52.5%)	Chi²=0.804, p=0.370
Focal slowing	38 (29.5%)	19 (27.9%)	19 (31.2%)	Chi²=0.159, p=0.690
Epileptic discharges	10 (7.8%)	4 (5.9%)	6 (9.8%)	p_Fisher_=0.516
MRI findings, *n (%)* n=141					
Pathological MRI overall	109 (77.3%)	57 (80.3%)	52 (74.3%)	Chi²=0.722, p=0.395
Limbic encephalitis (medio-temporal hyperintensities)	32 (22.7%)	14 (19.7%)	18 (25.7%)	Chi²=0.722, p=0.395
Other encephalitis (demyelinating/inflammatory lesions in multifocal areas)	19 (13.5%)	11 (15.5%)	8 (11.4%)	Chi²=0.499, p=0.480
Other pathological lesions (e.g. stroke, SAE)	33 (23.4%)	15 (21.1%)	18 (25.7%)	Chi²=0.414, p=0.520
Atrophy	27 (19.2%)	14 (19.7%)	13 (18.6%)	Chi²=0.030, p=0.863
Non-specific lesions	25 (17.7%)	13 (18.3%)	12 (17.1%)	Chi²=0.033, p=0.856
Pathological contrast enhancement n=117	19 (16.2%)	11 (18.3%)	8 (14.0%)	Chi²=0.397, p=0.529
Unremarkable MRI	32 (22.7%)	14 (19.7%)	18 (25.7%)	
FDG-PET findings, *n (%)* n=127					
Pathological FDG-PET overall	113 (89.0%)	50 (86.2%)	63 (91.3%)	Chi²=0.835, p=0.361
Pathological hypermetabolism overall	58 (45.7%)	27 (46.6%)	31 (44.9%)	Chi²=0.034, p=0.855
Pathological hypometabolism overall	61 (48.0%)	30 (51.7%)	31 (44.9%)	Chi²=0.583, p=0.445
Limbic encephalitis (hypermetabolism in the temporal lobes)	47 (37.0%)	20 (34.5%)	27 (39.1%)	Chi²=0.292, p=0.589
Other encephalitic pattern	21 (16.5%)	12 (20.7%)	9 (13.0%)	Chi²=1.335, p=0.248
Neurodegenerative pattern	12 (9.5%)	7 (12.1%)	5 (7.3%)	Chi²=0.857, p=0.355
Other pathological findings (e.g., medication effects)	59 (46.5%)	26 (44.8%)	33 (47.8%)	Chi²=0.114, p=0.736
Unremarkable FDG-PET	14 (11.0%)	8 (13.8%)	6 (8.7%)	

AE, autoimmune encephalitis; CSF, cerebrospinal fluid; EEG, electroencephalography; e.g., for example; FDG-PET, 18-F-fluor-desoxy-glucose-positron-emmission-tomography; IgG, immunglobulin G; l, litre; mg, milligramme; µl, microlitre; MRI, magnetic resonance imaging; mRS, modified Ranking Scale; n, number; OCB, oligoclonal bands; ref., reference; SAE, Subcortical leukoencephalopathy; SD, standard deviation; y, years.

Significant results are typed in bold.

### Immunotherapies and therapy response

The overall proportion of patients receiving immunotherapies was high in both groups, but significantly lower in patients with seronegative AE (53 (73.6%) *vs* 66 (86.8%) seropositive cases, p = 0.043). With regards to specific immunotherapies, seronegative AE patients were less frequently treated with intravenous immunoglobulins (IVIG, 7 (9.7%) *vs* 22 (29.0%), p = 0.003), rituximab (7 (9.7%) *vs* 20 (26.3%), p = 0.009), and azathioprine (8 (11.1%) *vs* 18 (23.7%), p = 0.045). There was also a trend towards seronegative AE patients being less frequently treated with high dose glucocorticoids (50 (69.5%) *vs* 63 (82.9%), p = 0.054). Of note, the proportion of patients being treated with therapeutic plasmapheresis was not statistically different between the groups (26 (31.1%) *vs* 32 (42.1%), p = 0.455). All patients with an underlying malignancy received oncological therapy according to current guidelines.

**Figure 1:**
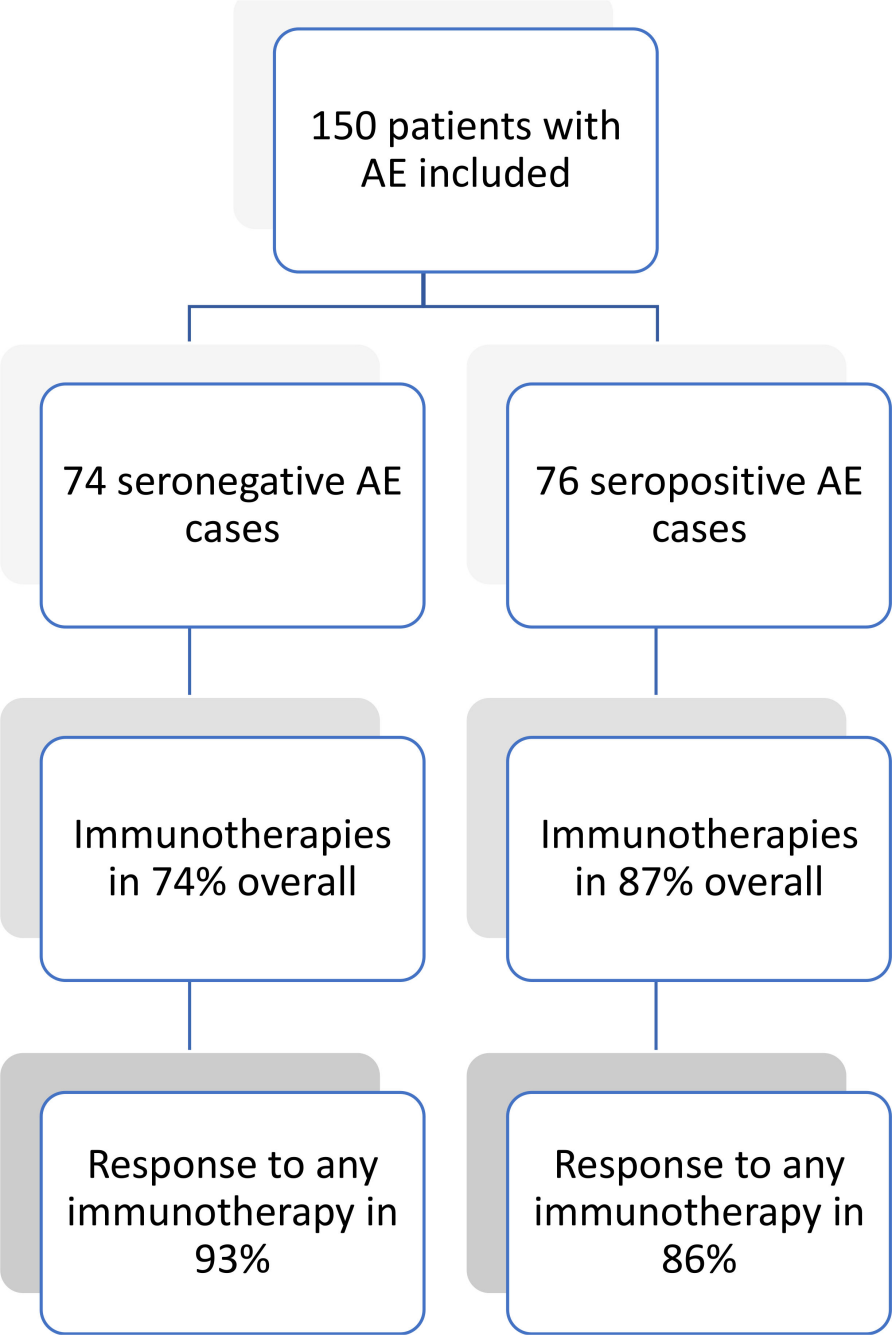
Therapy response in patients with seronegative and seropositive AE. AE, autoimmune encephalitis.

Therapy response on general impression by the treating physician was high, and not different between the groups (49 (92.5%) *vs* 57 (86.4%), p = 0.290) with most patients in both groups responding to first-line immunotherapies (44 (88.0%) *vs* 50 (80.7%), p = 0.292). However, there was a trend towards a longer length of hospital stay in the seronegative group (47.6 ± 52.3 *vs* 34.8 ± 36.4 days, p = 0.090). There was also a trend towards fewer patients having a favorable outcome (mRS 0-2) at discharge in the group of patients with seronegative AE (31 (43.1%) *vs* 44 (57.9%), p = 0.071). This difference became significant at last follow-up (33 (45.8%) *vs* 52 (68.4%) with mRS 0-2, p = 0.005). However, in both groups the proportion of patients with a favorable mRS of 0-2 was twice as high at last follow-up as compared to baseline (seronegative: 33 (45.8%) at last follow-up *vs* 16 (21.9%) at baseline; seropositive: 52 (68.4%) at last follow-up *vs* 25 (32.9%) at baseline). However, for the comparison of longer-term outcome, mean follow-up time was significantly shorter in seronegative AE patients (15.3 ± 24.9 *vs* 24.3 ± 28.1 months, p = 0.007). Three patients from the seropositive group (two during the acute phase, one during longer-term follow-up) and one patient from the seronegative group died, the latter also during the acute phase. **Figure 1** shows frequency of treatment and therapy response on general impression in both groups. Further details on immunotherapies, and all clinical outcomes are depicted in **Table 3**.

**Table 3:** Immunotherapies and outcome of patients with seronegative and seropositive AE.

	All n=150		Seronegative AE n=74	Seropositive AE n=76	p value (seronegative *vs*. seropositive)	
Immunotherapies, *n (%)* n=148						
Any immunotherapy	119 (80.4%)		53 (73.6%)	66 (86.8%)	**Chi²=4.108, p=0.043**
1^st^ line immunotherapies					
Glucocorticoids	113 (76.4%)		50 (69.4%)	63 (82.9%)	**Chi²=3.704, p=0.054**
Intravenous immunoglobulins	29 (19.6%)		7 (9.7%)	22 (29.0%)	**Chi²=8.674, p=0.003**
Therapeutic plasma exchange/Immunoadsorption	58 (39.2%)		26 (31.1%)	32 (42.1%)	Chi²=0.557, p=0.455
2^nd^ line immunotherapies						
Rituximab	27 (18.2%)		7 (9.7%)	20 (26.3%)	**Chi²=6.826, p=0.009**
Cyclophosphamide	3 (2.0%)		1 (1.4%)	2 (2.7%)	p_Fisher_=1.000
Other immunotherapies						
Azathioprine	26 (17.6%)		8 (11.1%)	18 (23.7%)	**Chi²=4.036, p=0.045**
Methotrexate	3 (2.0%)		3 (4.2%)	0 (0.0%)	p_Fisher_=0.113
Mycophenolate mofetil	1 (0.7%)		0 (0.0%)	1 (1.3%)	p_Fisher_=1.000
Bortezomib	0 (0.0%)		0 (0.0%)	0 (0.0%)	
Therapy response on general impression at any time point following immunotherapies, n (%)						
Response to any therapy n=119	106 (89.1%)	49 (92.5%)		57 (86.4%)	Chi²=1.120, p=0.290
Response to 1^st^ line immunotherapies n=112	94 (83.9%)	44 (88.0%)		50 (80.7%)	Chi²=1.110, p=0.292
Response to 2^nd^ line immunotherapies n=53	27 (50.9%)	8 (57.1%)		19 (48.7%)	Chi²=0.293, p=0.589
Response to other immunotherapies n=53	16 (30.2%)	6 (42.9%)		10 (25.6%)	p_Fisher_=0.311
Length of hospital stay in days, *mean (range, SD)* n=144	41.1 (0-335; 45.3)	47.6 (5-335; 52.3)		34.8 (0-217; 36.4)	**T=1.708,** **p_t-test_=0.090**
Outcome at discharge n=148						
General impression						
Improved, *n (%)*	113 (76.4%)	57 (79.2%)		56 (73.7%)	Chi²=0.615, p=0.433
Unchanged, *n (%)*	29 (19.6%)	12 (16.7%)		17 (22.4%)	Chi²=0.763, p=0.382
Worse, *n (%)*	6 (4.1%)	3 (4.2%)		3 (4.0%)	p_Fisher_=1.000
Outcome on modified Rankin Scale (mRS)						
mRS, *mean (range, SD)*	2.6 (0-6; 1.1)	2.7 (0-6; 1.1)		2.5 (1-6; 1.0)	T=1.339, p_t-test_=0.183
mRS 0-2, *n (%)*	75 (50.7%)	31 (43.1%)		44 (57.9%)	**Chi²=3.257, p=0.071**
mRS 3-6, *n (%)*	73 (49.3%)	41 (56.9%)		32 (42.1%)	
mRS improvement (≥ 1 point), n (%)	75 (50.7%)	38 (52.8%)		37 (48.7%)	Chi²=0.248, p=0.619
mRS worsening (≥ 1 point), n (%)	5 (3.4%)	2 (2.8%)		3 (4.0%)	p_Fisher_=1.000
Outcome at last follow-up n=148						
Duration till last follow-up in months from hospital admission, *mean (range, SD)*	19.9 (0-131; 27.7)	15.3 (0-131; 24.9)		24.3 (0-119.3; 28.1)	**Z=-2.716, p_MWU_=0.007**
General impression						
Improved, *n (%)*	111 (75.0%)	54 (75.0%)		57 (75.0%)	Chi²=0.000, p=1.000
Unchanged, *n (%)*	22 (14.9%)	12 (16.7%)		10 (13.2%)	Chi²=0.360, p=0.549
Worse, *n (%)*	15 (10.1%)	6 (8.3%)		9 (11.8%)	Chi²=0.500, p=0.480
Outcome on modified Rankin Scale (mRS)						
mRS, *mean (range, SD)*	2.3 (0-6; 1.3)	2.6 (0-6; 1.1)		2.0 (0-6; 1.4)	**Z=-3.290, p_MWU_=0.001**
mRS 0-2, *n (%)*	85 (57.4%)	33 (45.8%)		52 (68.4%)	**Chi²=7.716, p=0.005**
mRS 3-6, *n (%)*	63 (42.6%)	39 (54.2%)		24 (31.6%)	
mRS improvement (≥ 1 point), *n (%)*	90 (60.8%)	42 (58.3%)		48 (63.2%)	Chi²=0.361, p=0.548
mRS worsening (≥ 1 point), *n (%)*	14 (9.5%)	5 (6.9%)		9 (11.8%)	Chi²=1.036, p=0.309

AE, autoimmune encephalitis; mRS, modified Ranking Scale; MWU, Mann-Whitney U test; n, number; SD, standard deviation.

Significant results are typed in bold.

## Discussion

This study comprehensively describes use and response to immunotherapies as well as clinical features of patients with seronegative AE in comparison to seropositive cases. The main findings were that the vast majority of patients in both groups received immunotherapies (74% seronegative *vs* 87% seropositive), and showed relevant improvement (93% seronegative *vs* 86% seropositive). A favorable outcome (i.e., mRS 0-2) at last follow-up was achieved in 57% of cases (46% seronegative *vs* 68% seropositive). Despite a statistical difference between seronegative and seropositive AE patients with regards to achieving mRS 0-2, in both groups these numbers were twice the baseline value (22% seronegative *vs* 33% seropositive).

### Patient characteristics and paraclinical findings

Based on our data and another study with similar inclusion criteria (15), the frequency of seronegative AE equaled that of seropositive cases in our tertiary care university hospital, which contrasts previous prevalence data indicating a considerably lower frequency of seronegative AE (1). Differences between study populations, methods in data acquisition (hospital-based *vs* population-based), and an increasing awareness towards the diagnosis over time have to be taken into account. The high proportion of seronegative cases in our cohort emphasizes the importance of identifying these patients, since the majority benefitted from immunotherapies. Even though some clinical features (epileptic seizures and focal neurological deficits) were less frequent in seronegative patients in our study, these patients are usually regarded as clinically indistinguishable from seropositive cases (5, 15, 16). Furthermore, there were no specific findings differentiating seronegative and seropositive AE cases using CSF analysis, EEG, MRI or FDP-PET imaging as reported previously (5, 8, 15–17). Hence, in the acute clinical setting, when antibody results are pending, or during further course, when all antibody results turn out to be negative, seronegative and seropositive AE patients seem to be mostly indistinguishable based on clinical and paraclinical features, yet therapeutic decisions have to be made.

In comparison to two previous studies, patients with seronegative AE in our study less frequently had seizures (36% in our study *vs* 81% and 65%, respectively) (16, 18), psychiatric symptoms (59% in our study) were either less frequent (76%) (18) or identical (16), and memory problems (76% in our study) were either less frequent (93%) (18) or more frequent (62%) (16). These differences are most likely due to different patient populations as well as different inclusion criteria. Of note, all three categories of symptoms were most frequent in the study by Lee et al., which might be attributable to the prospective study design (18), whereas our study and the study by Gastaldi et al. were retrospective (16).

### Immunotherapies and therapy response

As stated above, the vast majority of patients with seronegative AE in our study received immunotherapies, which is in line with previous publications (16–19). Relative frequency of various immunotherapies were in line with other studies with glucocorticoids and other first-line immunotherapies (IVIGs and plasmapheresis) being given to most of the patients, regardless whether they are seronegative (17–19) or seropositive (7, 16).

With regards to therapeutic response rates reported previously in patients with AE, these were generally high, yet naturally revealed some variations dependent on patients included, outcome parameter applied and time point analyzed. The thus far largest study included 577 seropositive patients with NMDAR encephalitis only (7), of whom 472 (82%) received first-line therapies. Of these, 251 (53%) showed improvement on the mRS within four weeks. With regards to seronegative cases only, the thus far largest study that included 147 patients with exclusively seronegative AE (117 with antibody-negative probable AE, 23 with seronegative limbic encephalitis, and 7 with acute demyelinating encephalomyelitis) was performed by Lee and colleagues (18). In this study, a favorable 2-year outcome, defined as a mRS 0-2, was achieved in 57%, which is roughly within the order of magnitude of our data. However, direct comparison is limited due to divergent inclusion criteria, and since in our study patients were less severely affected at baseline (median mRS 5.0 in the study by Lee et al. *vs* a mean mRS 3.3 in our study), follow-up time was shorter (2 years in the study by Lee et al. *vs* a mean of 19.9 months in our study), time till treatment initiation longer (median 8.0 days in the study by Lee et al. *vs* a mean of 14 weeks between primary manifestation and hospital admission in our study), and treatment less aggressive, i.e., Lee et al. treated 79% with rituximab (*vs* only 9.7% in our study), 40% with tocilizumab (not performed on our cohort), and 10% with cyclophosphamide (*vs* 1.4%). The high rate of seronegative AE patients being treated with rituximab in the study by Lee et al. at first glance seems counterintuitive (18). However, the high efficacy indicates a significant role of B cells in seronegative cases. Speculatively, these patients have antibodies against yet to be identified antigens or pathophysiologically antibody-independent B cell immunity plays a role (20–22). These data challenge current guidelines recommending use of rituximab preferably in seropositive AE (9), and could encourage treating physicians to consequently escalate therapy also in seronegative AE patients.

In the thus far only other study directly comparing treatment efficacy in patients with seronegative and seropositive AE by Gastaldi et al. the majority of patients (110/118; 93.2%) received immunotherapies, and showed clinical improvement (84.5%) (16). Yet, in contrast to our data seronegative cases responded less frequently than seropositive patients (65.6% *vs* 92.3%), which was at least in part attributable to delays in treatment initiation as well as less patients receiving second-line immunotherapies (13.7% *vs* 39.5%) in this group (16).

Two studies focused on seronegative limbic encephalitis only, and included 11 and 28 patients, respectively (17, 19). Following immunotherapies, an improvement was achieved in 54% and – dependent on the outcome parameter applied – 11-48% of cases, which was considerably lower compared to 93% clinical improvement in our study including unselected seronegative patients, and also lower compared to 81% in another study including unselected seropositive (68%) and seronegative (32%) patients (1). Even though mode and frequency of various treatments were not generally different between these studies, a generally worse prognosis in limbic encephalitis has been presumed (17–19).

The other side of the coin with rapid initiation and escalation of immunotherapy, especially in seronegative AE cases, is the potential of misdiagnosis, and unnecessarily exposing patients to side effects. In a recent retrospective study, this related to 27% of all patients, who were first diagnosed with AE, and later identified to have other disease. However, the majority of these (77%) was not fulfilling diagnostic consensus criteria, and had diagnosis other than AE, e.g., functional neurological syndromes or psychiatric disorders (23). Hence, strict adherence to consensus criteria and exclusion of differential diagnosis would have avoided these misclassifications (5).

Our study has several limitations. First, due to the retrospective design, some data was missing in patients, and the length of follow-up was highly variable, notably shorter in seronegative AE patients. However, the majority received standardized diagnostic work-up with clinical as well as neuropsychological testing, CSF analysis, antibody testing in both serum and CSF, and imaging with both MRI and FDG-PET. Nonetheless, electronically recorded data were insufficient in the retrospective setting to calculate the CASE score (Clinical Assessment Scale in Autoimmune Encephalitis) during follow-up, which had been specifically developed as an objective measure to evaluate outcome in patients with AE (24). Instead, a qualitative and subjective measure (“general impression”) as well as the quantitative, but not AE-specific mRS were applied, since these were easily available and had been used by others (7, 16, 18). In addition, data on the premorbid status of our patients were insufficient. Hence, it might be that the higher mRS in the seronegative group at admission was due to a higher premorbid mRS. However, this is rather speculative. Second, not all patients (n = 32, 21%) were fulfilling consensus criteria (5). However, cases were extensively discussed, and still included if satisfying recent “best practice recommendations for diagnosis and acute management” of AE (9). These recommendations comprise a broader clinical spectrum in comparison to the consensus criteria, e.g. patients with cerebellitis or cerebellar degeneration and encephalomyelitis. According to these recommendations, CSF-restricted OCBs instead of pleocytosis (0.7% of our cases) and FDG-PET instead of MRI alterations (3.3% of our cases) might support an inflammatory etiology in the right clinical setting and after exclusion of alternative diagnosis. Additionally, patients fulfilling all criteria except for the subacute presentation within three months were included (6.7%). The reason, why a significant proportion of our patients was not fulfilling this criterion was potentially due to a high proportion of patients with a pure psychiatric syndrome, who tend to not present within the acute stage of the disease. Finally, a significant number of patients (10.7%) had antibodies against unknown neuronal antigens, which according to the criteria “strongly support the diagnosis of autoimmune encephalitis” (5). According to a recent publication analyzing “autoimmune encephalitis criteria in clinical practice”, in 538 patients diagnosed at the Mayo clinic, 177 (33%) were not fulfilling consensus criteria, a significant number having inflammatory CSF alterations other than pleocytosis (e.g. OCBs), presenting outside the 3-months-interval or having antibodies against unknown neuronal antigens (25). Third, due to the monocentric design the question of generalizability arises. However, our tertiary care university hospital has a large catchment area of approximately 2,500,000 inhabitants and the inclusion of both neurologic and psychiatric patients warrants inclusion of the majority if not all patients who had been diagnosed with AE in Southwestern Germany within a ten-year period. In addition, the monocentric design made sure, that all patients received identical diagnostic work-up and had access to the same therapeutic options. In this regard, previous studies showed good consistency with respect to glucocorticoids being used as first choice. However, the use of other therapeutic options (e.g., plasmapheresis, rituximab) showed great variability (1, 7, 16–19), probably due to personal preferences and experiences. Fourth, due to the continuous identification of novel target antigens (22), the classification of patients into the two categories seronegative and seropositive naturally changes over time. However, for reasons of consistency and comparability with previous studies we used definitions of currently available consensus criteria and guidelines (5, 9). Fifth, since the group of seronegative patients was presumably heterogenic, potentially including patients with low titer antibodies, antibodies against yet to be identified antigens, with cell-mediated immune processes, but also with non-immunogenic etiologies in some cases, expected therapy response also would be heterogenic. The high response rate to immunotherapies at first glance indicates an immunogenic pathophysiology in most cases. However, a clear diagnostic marker – as with antibodies in all seropositive cases – is lacking. Hence, improvement in some cases might also reflect spontaneous disease course or response to concomitant medication (e.g., anticonvulsants or neuroleptics), rather than immunotherapy response. Therefore, further research on the underlying pathophysiology is urgently needed. Presuming heterogenous pathophysiological processes in patients with seronegative AE, we decided not to subdivide patients with seropositive AE into those with antibodies against cell-surface and intracellular antigens. Since the latter are known to show poor response to immunotherapies, this would have introduced a relevant selection bias.

In conclusion, the vast majority of patients with seronegative AE benefitted from immunotherapies. Of note, treatment response was not different in comparison to seropositive cases. This indicates that international consensus criteria for the diagnosis of AE, that were deliberately not reliant on antibody results (5), and current guidelines (9) as well as rigorous exclusion of alternative diagnosis if an autoimmune etiology is presumed, seem to be sufficient in identifying patients benefitting from immunotherapies, regardless whether they are seronegative or seropositive. This is of particular clinical importance, since seronegative patients are otherwise clinically and paraclinically mostly indistinguishable from seropositive AE cases, and therapeutic decisions have to be made often before antibody results are available. For more reliable conclusions prospective multicenter studies would be necessary. The pathophysiological mechanisms underlying seronegative AE (e.g., novel neuronal antibodies or T-cell mediated processes) should be analyzed in parallel.

## Data availability statement

The original contributions presented in the study are included in the article/supplementary material. Further inquiries can be directed to the corresponding author.

## Ethics statement

The studies involving human participants were reviewed and approved by local ethics committee of the University of Freiburg. Written informed consent for participation was not required for this study in accordance with the national legislation and the institutional requirements.

## Author contributions

BB and DE were involved in the design and conceptualization of the study. SH had a major role in the acquisition of data. SH and KR performed statistical analysis. BB, SH and DE were involved in data analysis and interpretation. BB drafted the first version of the manuscript, which was then revised by all authors for intellectual content. All authors contributed to the article and approved the submitted version.
